# Supplementary material for: SARS-CoV-2 Delta Variant: Interplay between Individual Mutations and Their Allosteric Synergy
Source: Biomolecules. 2022 Nov 23;12(12):1742. doi: 10.3390/biom12121742 (PMC9775976; doi:10.3390/biom12121742)
Supplement: Supplementary file 1 [file biomolecules-12-01742-s001.zip › biomolecules-2021325-supplementary.pdf]

Supplementary Methods, Figures, and Tables

SARS-CoV-2 Delta Variant: Interplay between Individual Mutations and Their Allosteric Synergy

Kevin C. Chan [1,2], Yi Song [1], Zheng Xu [3], Chun Shang [1] and Ruhong Zhou [1,2,4,5,*]

1 Institute of Quantitative Biology, College of Life Sciences, Zhejiang University, Hangzhou 310058, China

2 Shanghai Institute for Advanced Study, Zhejiang University, 799 Dangui Road, Shanghai 201203, China

3 BirenTech Research, Shanghai 201112, China; zxu@birentech.com

4 Department of Chemistry, Columbia University, New York, NY 10027, USA

5 The First Affiliated Hospital, School of Medicine, Zhejiang University, Hangzhou 310058, China

* Correspondence: rhzhou@zju.edu.cn

Materials and Methods

MD simulations

The simulations were initiated from the crystal structures from Protein Data Bank (PDB) of RBD-ACE2 complex (PDB ID: 6M0J), RBD-B38 complex (PDB ID: 7BZ5), RBD--COVA2-39 complex (PDB ID: 7JMP), RBD--P2B-2F6 complex (PDB ID: 7BWJ), and RBD-CR3022 complex ((PDB ID: 6YLA). Modeller [1] was used to model missing residues and sidechains. Hydrogen atoms and protein chain terminus (capped with neutral groups acetyl and methyl amide) were added using Gromacs [2] pdb2gmx tool. All titratable residues were left in their dominant protonation state at pH 7.0. The systems were then solvated with TIP3P [3] water molecules and 0.15 M NaCl using Gromacs [2] tools. In all simulations, CHARMM36 forcefields [4,5] were used for protein, water, and salt molecules.

Simulations were performed on GPUs using Gromacs 2020.6 [2] for MD simulations. All simulations were performed with periodic boundary conditions and time steps of 2 fs. Bond lengths to hydrogen atoms were constrained with SHAKE [6]. Non-bonded interactions were cut off at 12.0 Å, and long-range electrostatics were computed using particle mesh Ewald (PME) [7] method with cubic interpolation. Systems were first minimized, annealed to 310 K in NVT ensemble and then equilibrated by stepwise releasing harmonic restraints on backbone of the protein molecules for a total of at least 10 ns. Production simulations were performed in NPT ensemble of 310 K and 1 bar with v-rescale [8] thermostat and Parrinello-Rahman [9] barostat in Gromacs. Multiple independent simulations were initiated from the final snapshot of the restrained equilibration. Otherwise specified, the first 100 ns of all the trajectories were discarded for analysis or snapshot picking.

FEP simulations

The simulations were initiated from snapshots of independent MD simulations. At least two snapshots (replicas) were obtained for each system (except for the RBD-CR3022 complex). When multiple snapshots were used for FEP simulations, number of independent simulations for each replica would be approximately the same to avoid biased results. System setups were similar to that for the MD simulations except VMD [10] plugins were used for adding water and salt molecules. CHARMM36 [4,5] forcefields were used for all the simulations.

Simulations were performed on GPUs using NAMD [11] version 3 with CUDA accelerations. All simulations were performed with periodic boundary conditions and time steps of 2 fs. Simulation parameters were similar to that in the MD simulations except Langevin thermostat [12] and barostat [13] were used to couple the temperature and pressure at 310 K and 1 bar. The dual topology was implemented using VMD [10] Mutator plugin. Each system was carefully mutated using **λ** increments of maximum 0.04, totaling 21 to 60 FEP windows depending on different mutation sets. Each window was run for 600 to 2000 ps depending on the convergence. Electrostatics were switched starting at **λ** = 0.1. Convergence for each window was assessed by comparing values across replicas. Error bars for free energy changes were standard errors otherwise specified.

To apply a free energy decomposition analysis to our FEP calculations, we employed the exponential-form approach. Such decomposition can be done through equations

$$\Delta G_{elec} \approx -\frac{1}{\beta}\ln\left(\frac{1}{N}\sum_{i=1}^{N} e^{-\beta\Delta V_{elec}}\right)$$

$$\Delta G_{vdw} \approx -\frac{1}{\beta}\ln\left(\frac{1}{N}\sum_{i=1}^{N} e^{-\beta\Delta V_{vdw}}\right)$$

, where N is the number of configurational samplings and ΔV_{elec} and ΔV_{vdw} are the differences in electrostatic and the van der Waals' interactions, respectively.

Analysis of trajectories

The buried area was computed by the following equation using solvent accessible surface area (SASA) calculations:

$$Buried\ Area = {(SASA_{RBD} + SASA_{ACE2} - SASA_{RBD+ACE2})}/{2}$$

VMD [10] was used for the SASA calculations with a water radius of 1.4 Å. Non-bonded interaction energies were computed using Gromacs [2] tools. Hydrogen bonds and salt bridges were computed using VMD [10] plugins. Averages and error bars were computed by discarding the first 100 ns of the MD simulations. Error bars shown were standard deviations.

Computation of water-mediated interactions

The residual hydration levels (Figure S9) were computed using in-house TCL script. First, all water molecules within 4 Å of the residue were counted for all the residues of the RBD and for all the frames of the MD or FEP simulations. Then, the average numbers of water molecules taken from the MD simulations were used as the reference numbers. These reference numbers were subtracted from the numbers calculated for each frame of the FEP simulations in order to obtain a difference in number of water molecules around a specific residue (i.e., the hydration level of the residue). Meanwhile, standard deviations (SDs) of the reference numbers were also calculated so that only if the difference was greater than (or smaller than) three SDs, it would be colored in blue (hydrated) or red (dehydrated) in Figure S9. To avoid over-amplification due to small SDs, all SDs of less than 1.0 were capped to 1.0. As a result, the change in hydration level would indicate at least three water molecules changes with respect to that in equilibrated MD simulations.

Hydrogen bonds were computed using VMD [10] HBonds plugin with the following geometric criteria: donor to acceptor distance less than 3.5 Å and donor-hydrogen-acceptor angle greater than 150°. A water-mediated interaction between a pair of residues is defined when both residues form hydrogen bonds with the same water molecule or with a pair of water molecules that in turn are also forming a hydrogen bond. Water-mediated interactions were computed for every frame of the MD simulations. However, for the FEP simulations, only the frames from last five alchemical states were considered in the statistics in Figure 3E, representing only the end state.

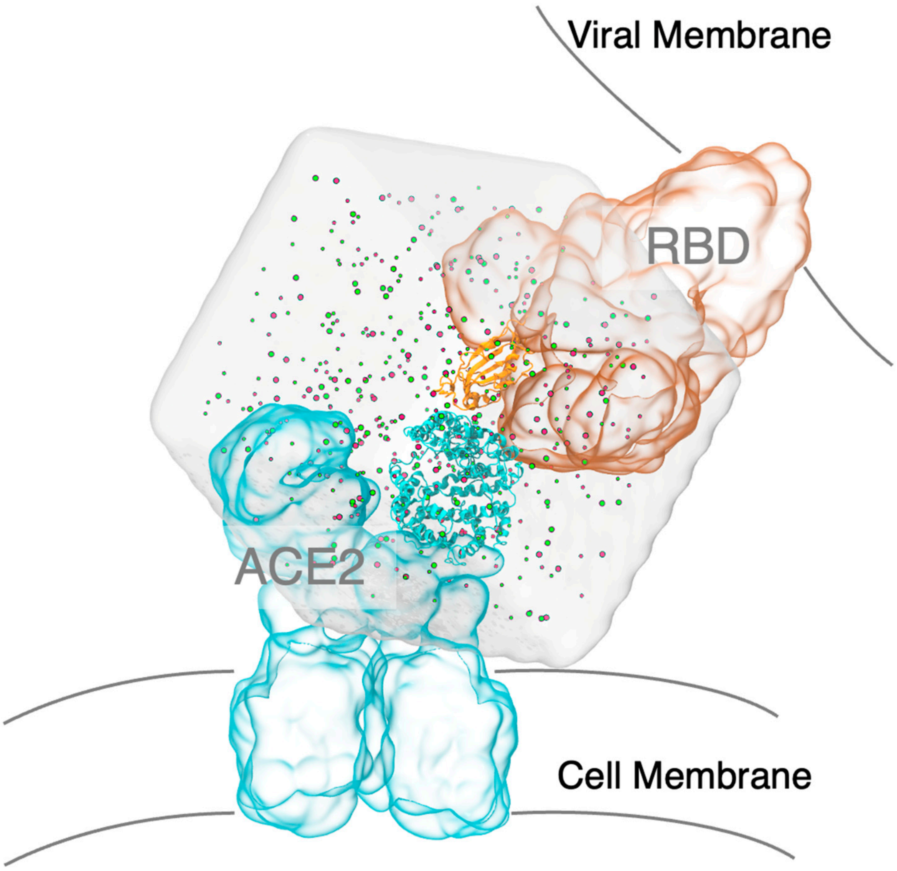

Figure S1. Schematics showing the simulation setup of viral RBD (orange) binding to human ACE2 (cyan). Water molecules were shown as transparent box with ions colored in red for sodium and green for chloride. Only the simulated part of the proteins was rendered in cartoon and the rest were in transparent surfaces.

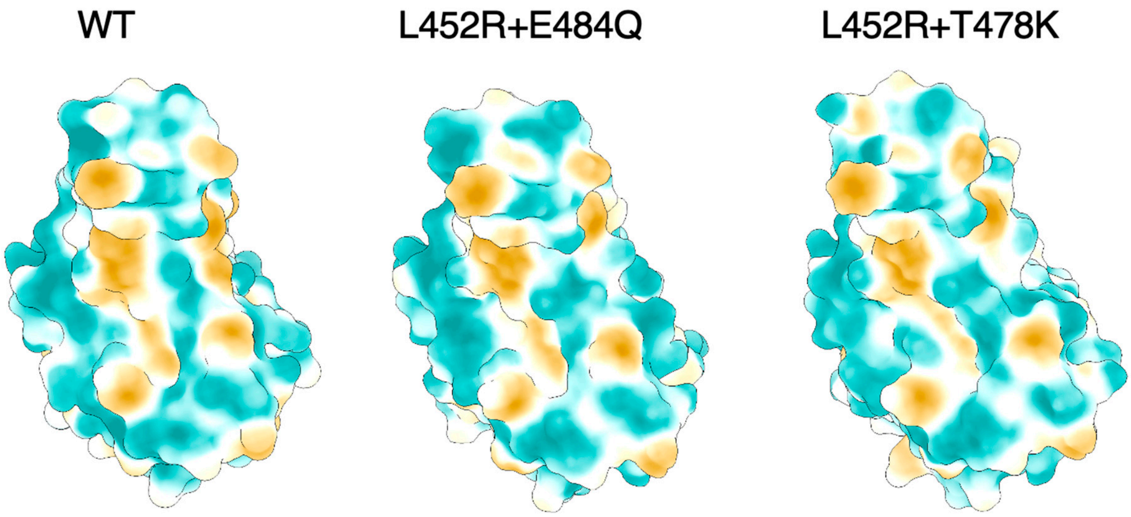

Figure S2. Molecular surface of WT and mutated RBDs. Hydrophobic and hydrophilic regions were colored in yellow and blue respectively.

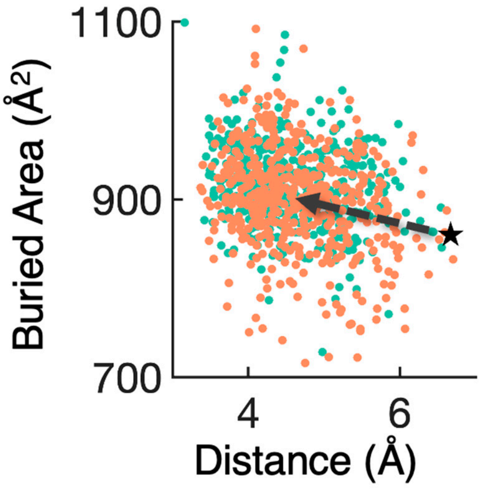

Figure S3. Distribution of distance between Gln493 and ACE2 correlated with the buried area between biomolecules. Asterisk indicates the crystal structure.

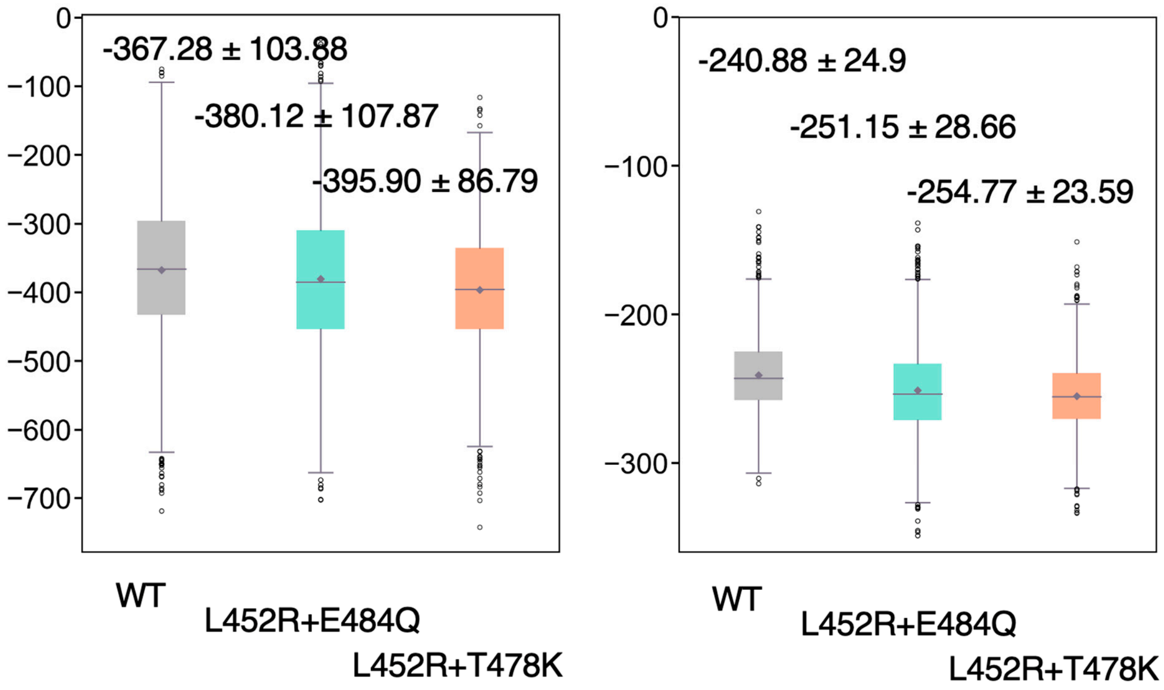

Figure S4. Interaction energy between WT or mutated RBDs and ACE2 decomposed into electrostatic (left) and Van der Waals (right). In the box plot, horizontal line indicates the average, grey dot indicates the median, and the box indicates the upper and lower quartiles.

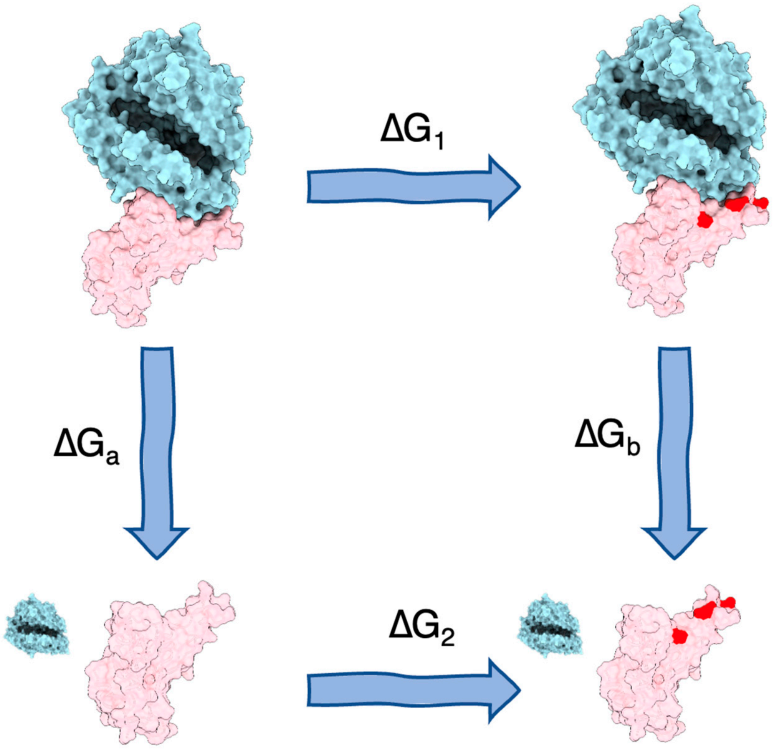

Figure S5. Designed thermodynamic cycle for the FEP calculations.

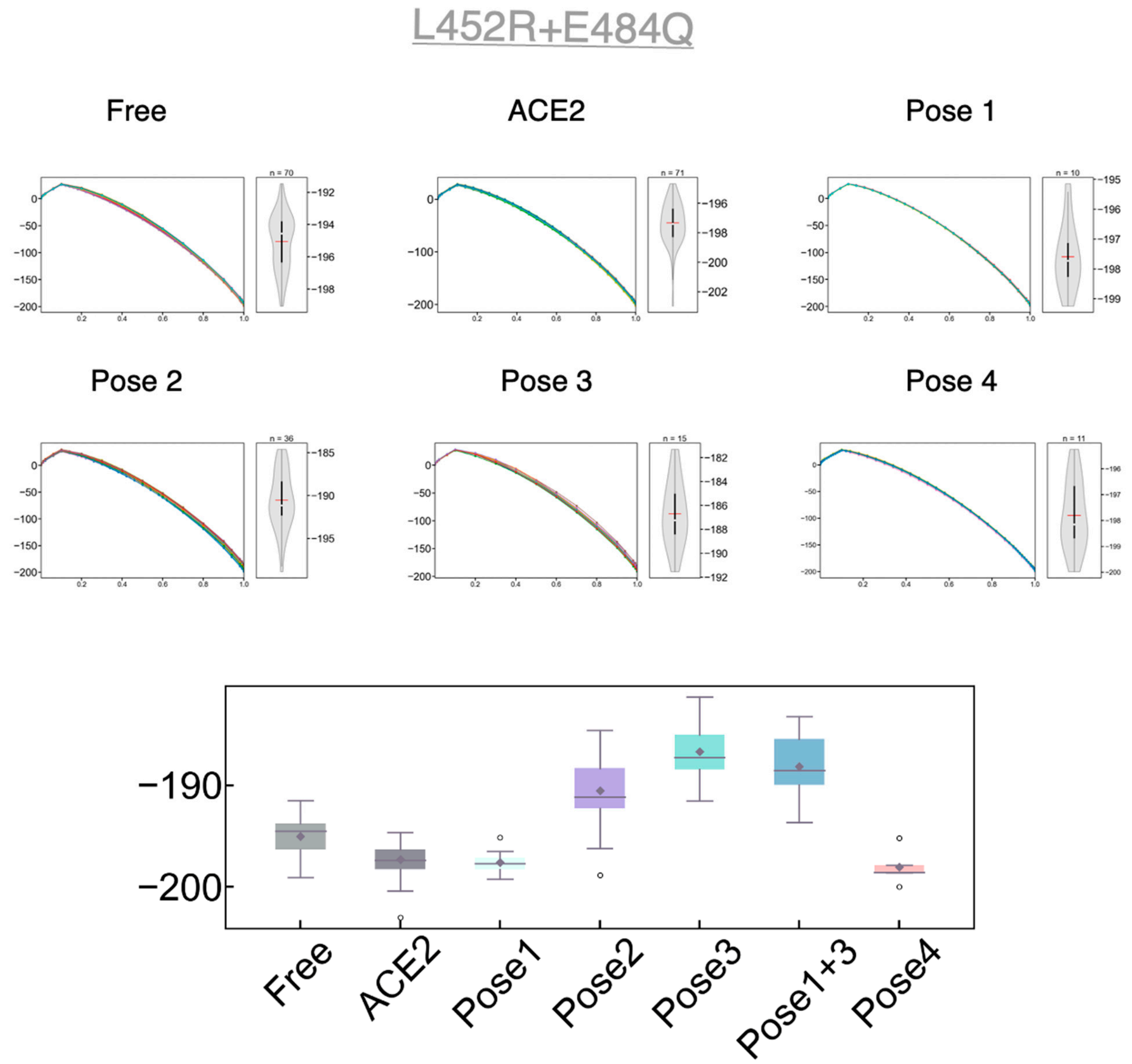

Figure S6. Free energy changes from FEP calculations of mutations L452R+E484Q for RBD alone (free state) or in complex with ACE2 or mAbs (Pose 1-4). The violin and box plots include all the data from the calculations. All free energies are in kcal/mol. In the violin plot, red line indicates the average, white dot indicates the median and black box indicates the upper and lower quartiles. Number of replicas are printed on the top of the violin plots.

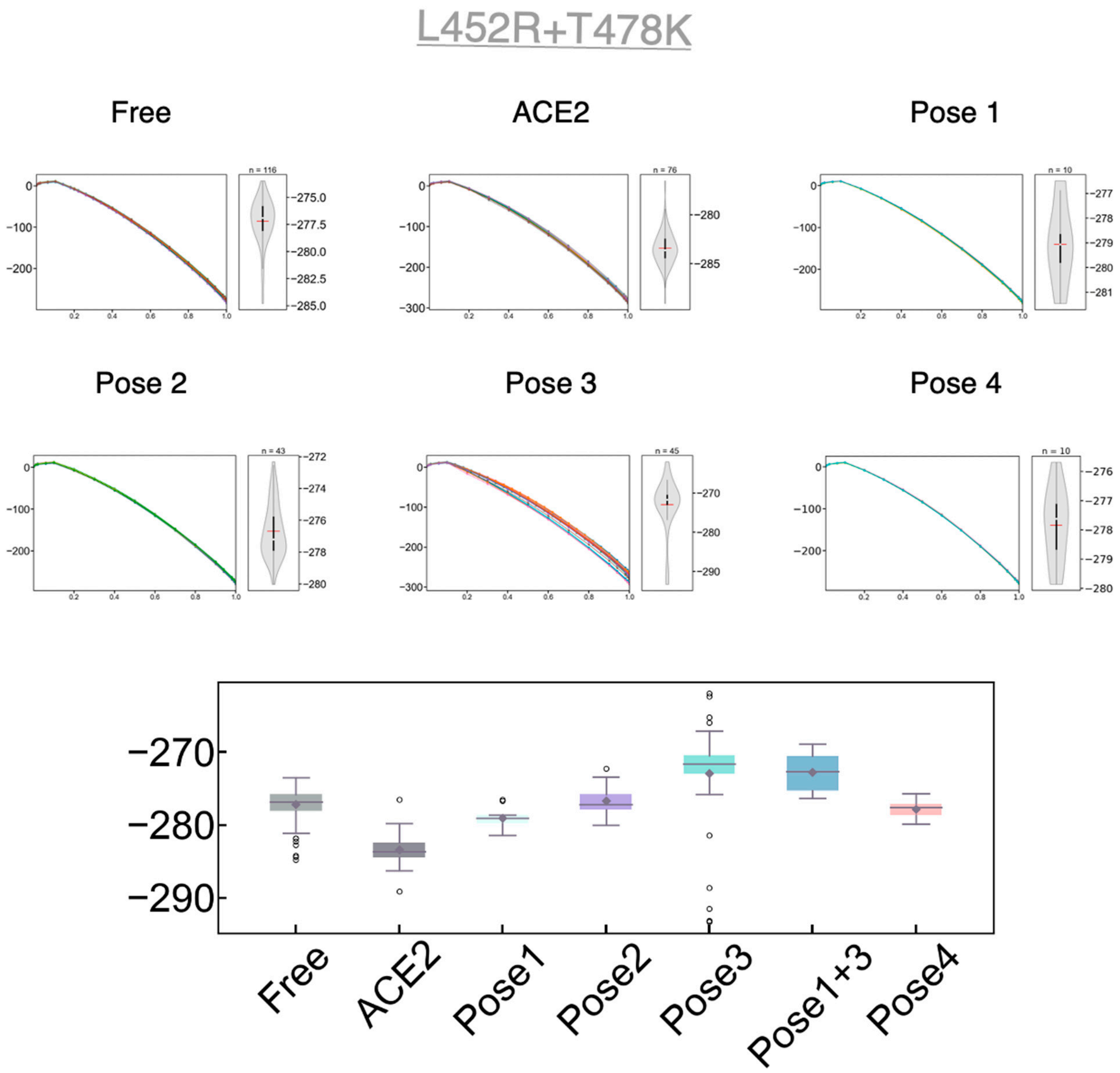

Figure S7. Free energy changes from FEP calculations of mutations L452R+T478K for RBD alone (free state) or in complex with ACE2 or mAbs (Pose 1-4). The violin and box plots include all the data from the calculations. All free energies are in kcal/mol. In the violin plot, red line indicates the average, white dot indicates the median and black box indicates the upper and lower quartiles. Number of replicas are printed on the top of the violin plots.

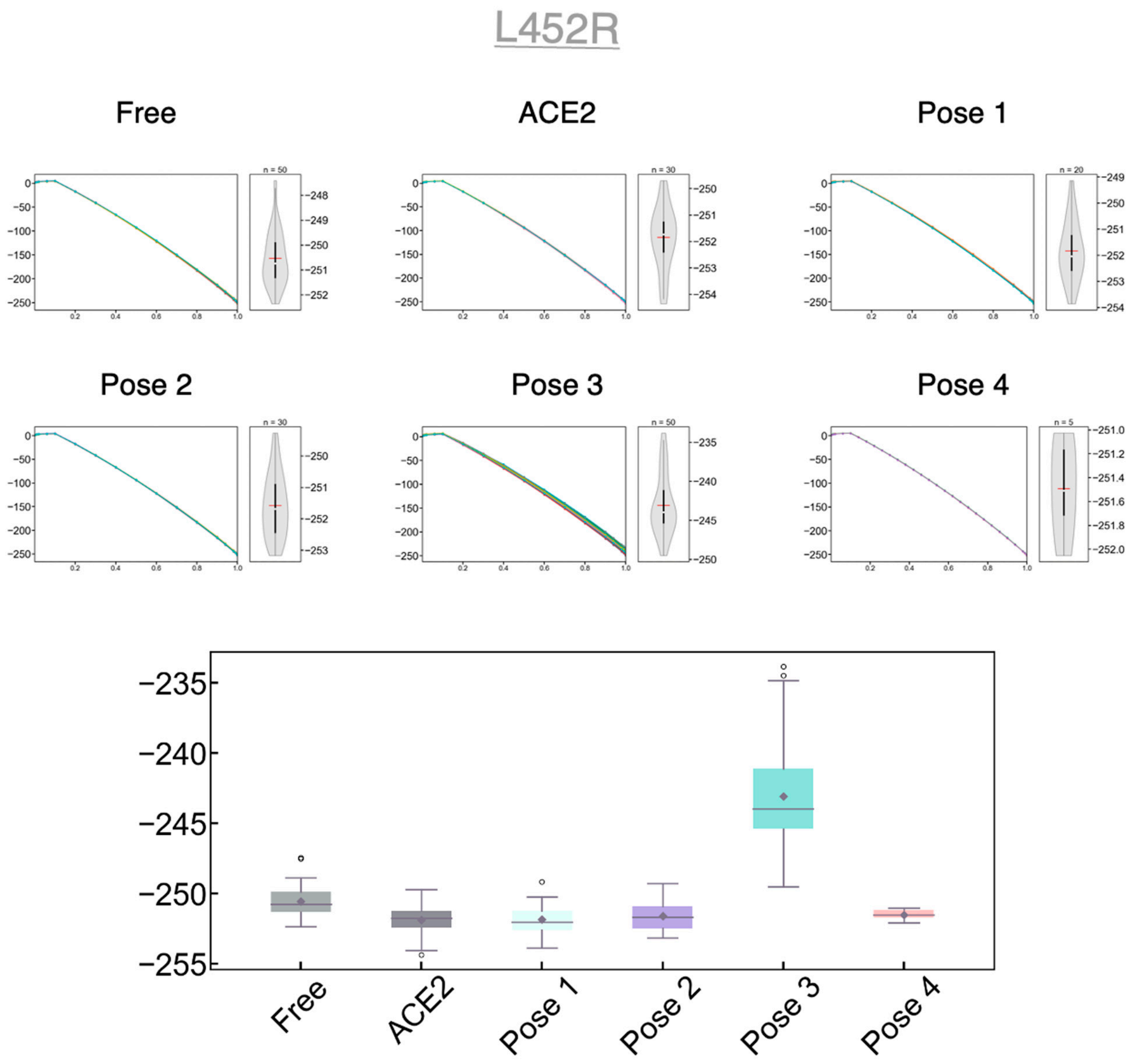

Figure S8. Free energy changes from FEP calculations of mutations L452R for RBD alone (free state) or in complex with ACE2 or mAbs (Pose 1-4). The violin and box plots include all the data from the calculations. All free energies are in kcal/mol. In the violin plot, red line indicates the average, white dot indicates the median and black box indicates the upper and lower quartiles. Number of replicas are printed on the top of the violin plots.

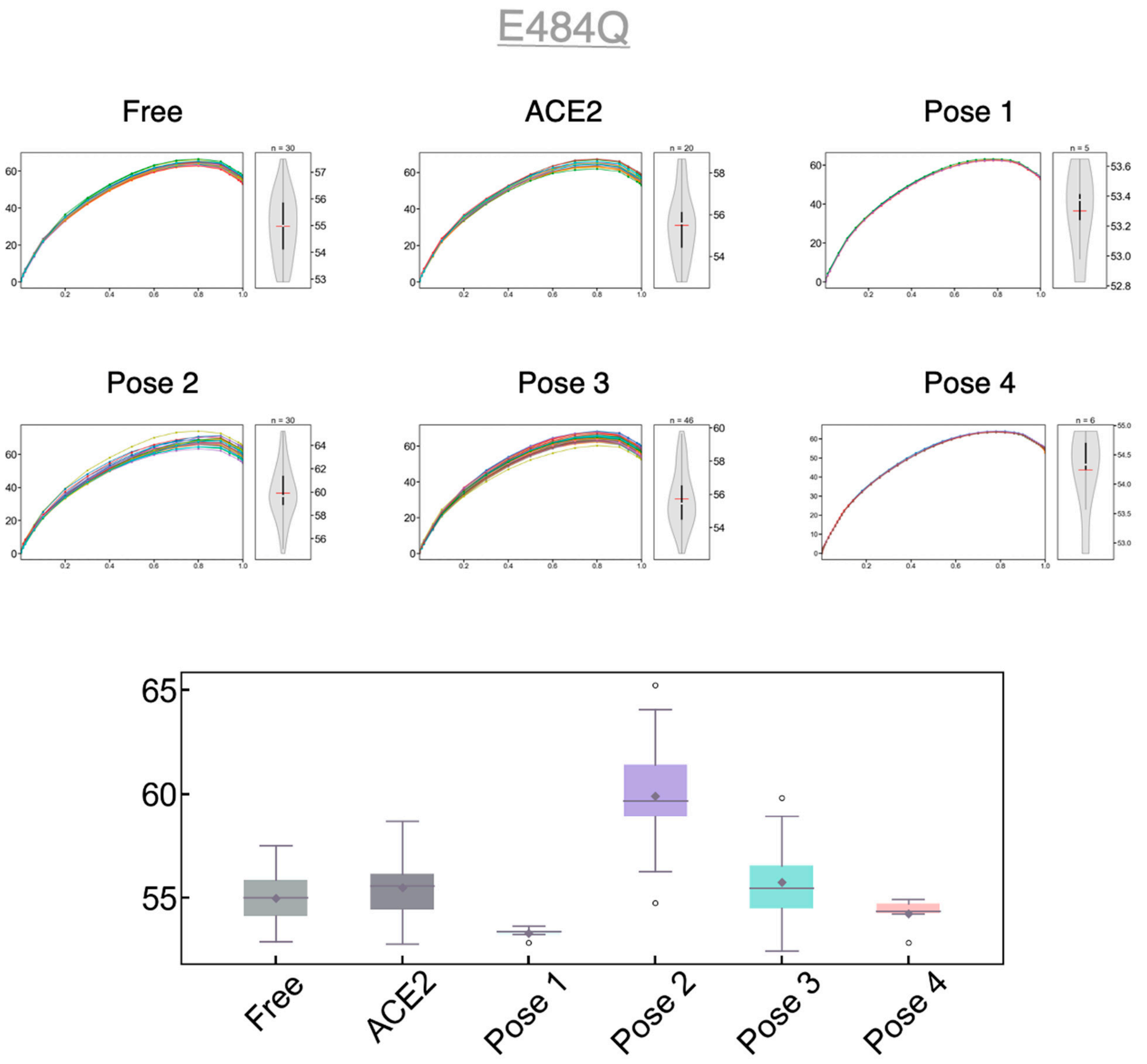

Figure S9. Free energy changes from FEP calculations of mutations E484Q for RBD alone (free state) or in complex with ACE2 or mAbs (Pose 1-4). The violin and box plots include all the data from the calculations. In the violin plot, red line indicates the average, white dot indicates the median and black box indicates the upper and lower quartiles. Number of replicas are printed on the top of the violin plots.

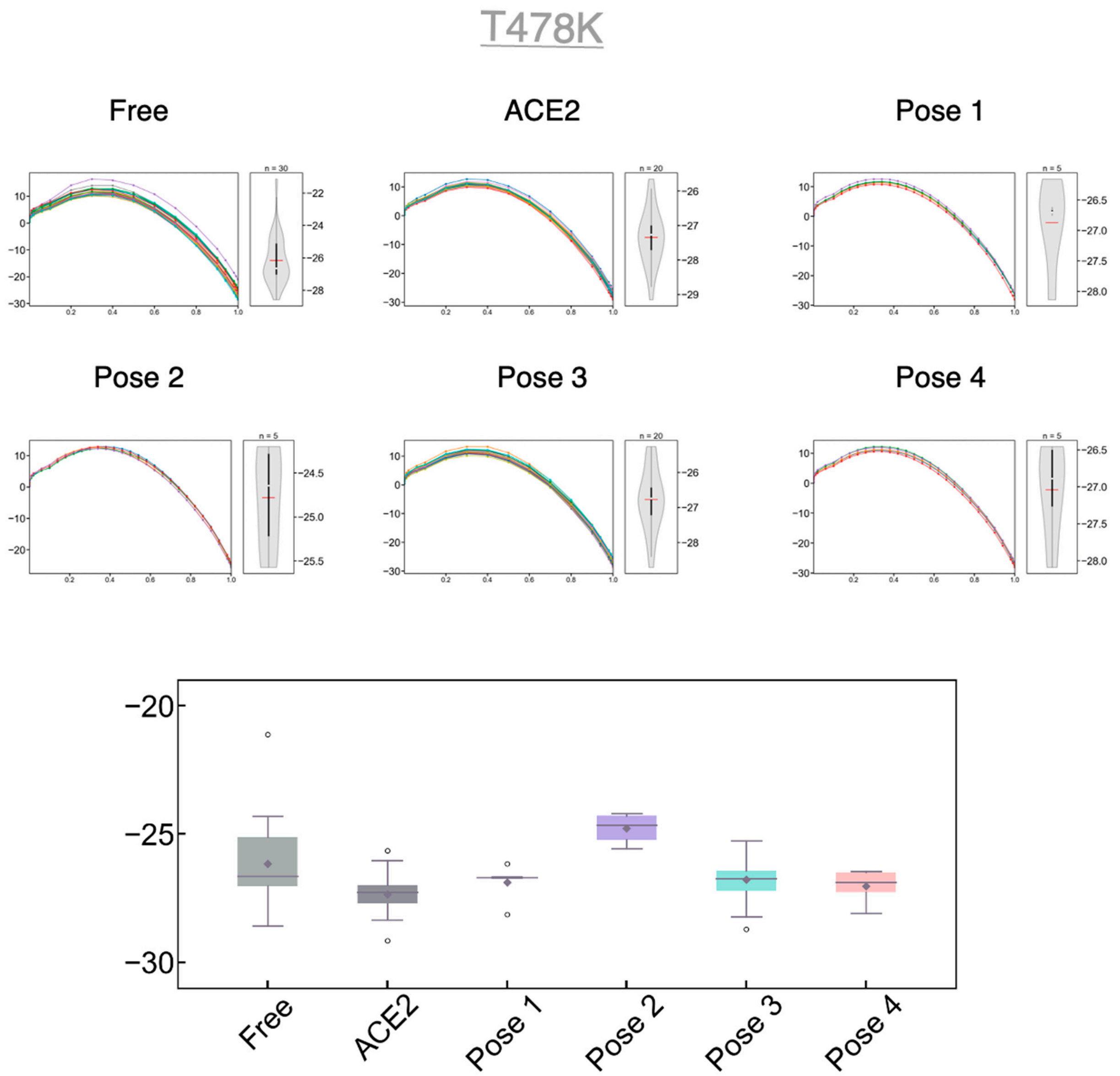

Figure S10. Free energy changes from FEP calculations of mutations T478K for RBD alone (free state) or in complex with ACE2 or mAbs (Pose 1-4). The violin and box plots include all the data from the calculations. All free energies are in kcal/mol. In the violin plot, red line indicates the average, white dot indicates the median and black box indicates the upper and lower quartiles. Number of replicas are printed on the top of the violin plots.

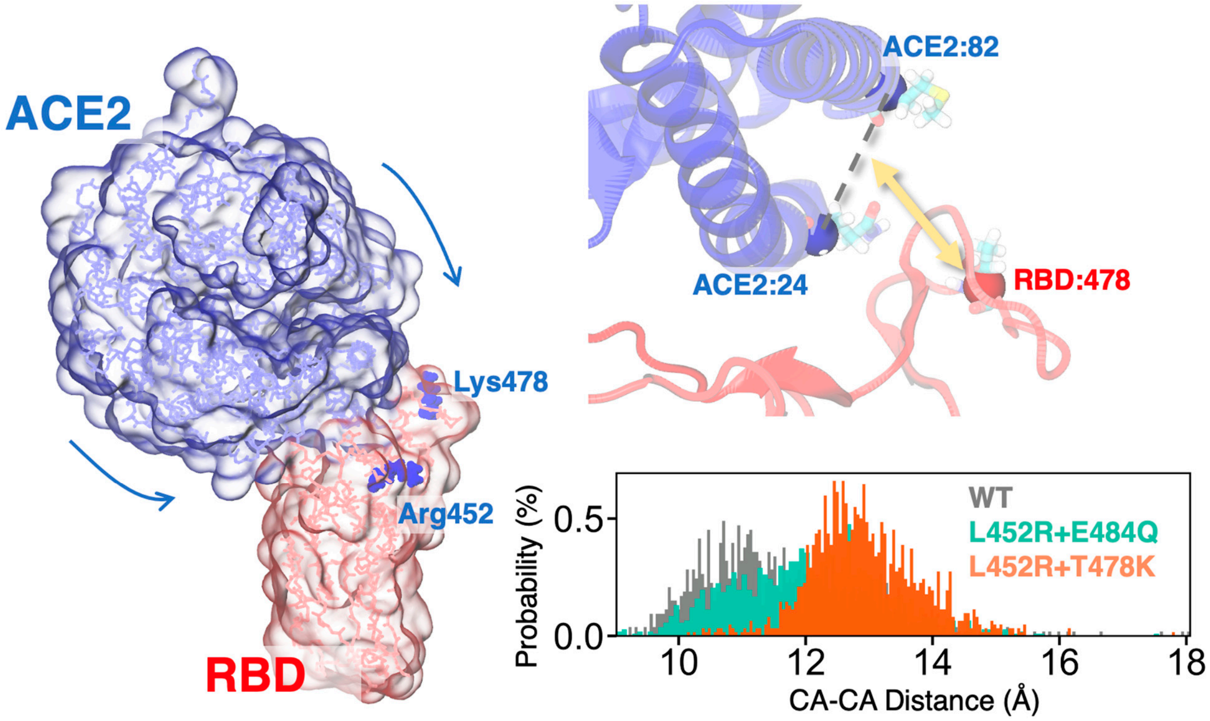

Figure S11. (A) RBD_{mut2} (L452R+T478K) in complex with ACE2 with Arg452 and Lys478 shown as blue sticks. (B) Distance between residue 478 of RBD and the adjacent helices of ACE2 in the complex.

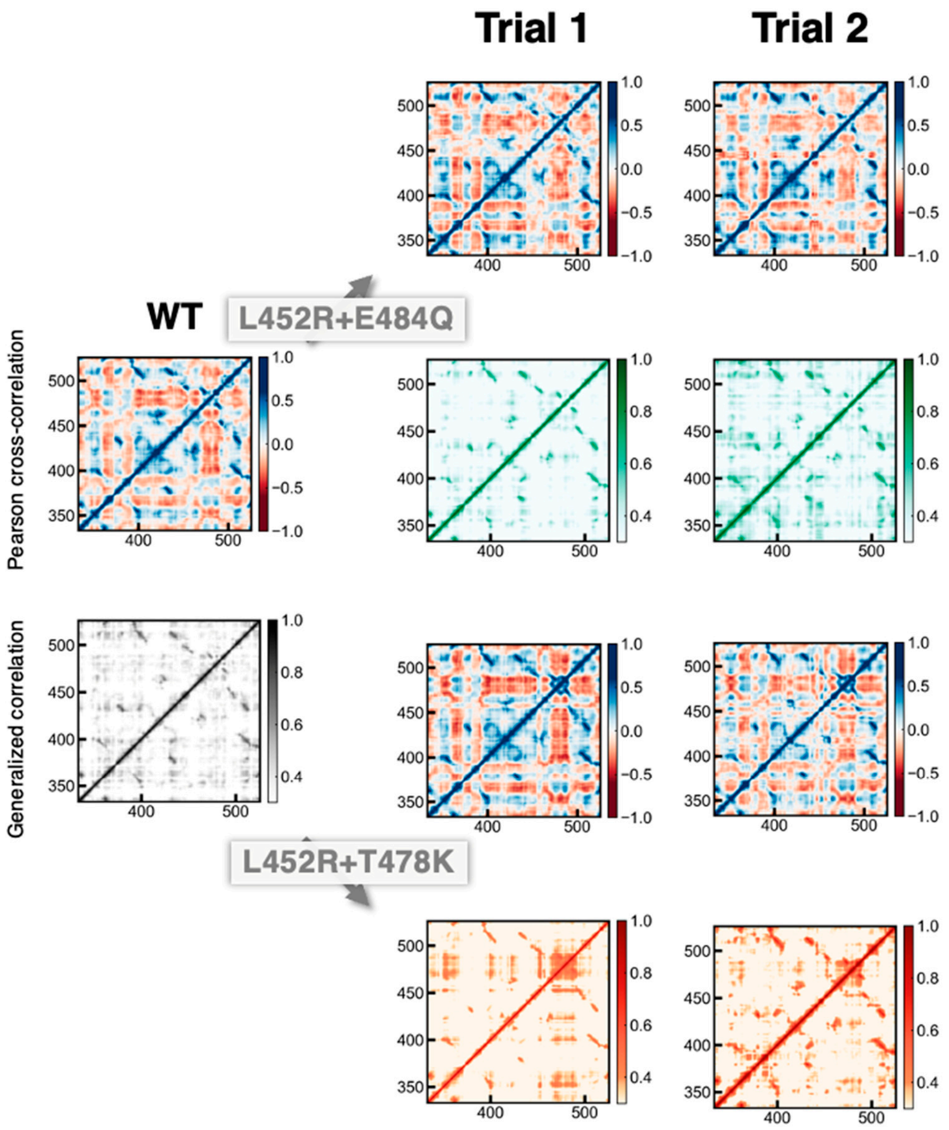

Figure S12. Pearson cross-correlation (above in the row) and generalized correlation (below in the row) of WT or mutated RBDs in complex with ACE2 obtained from two trials of MD simulations.

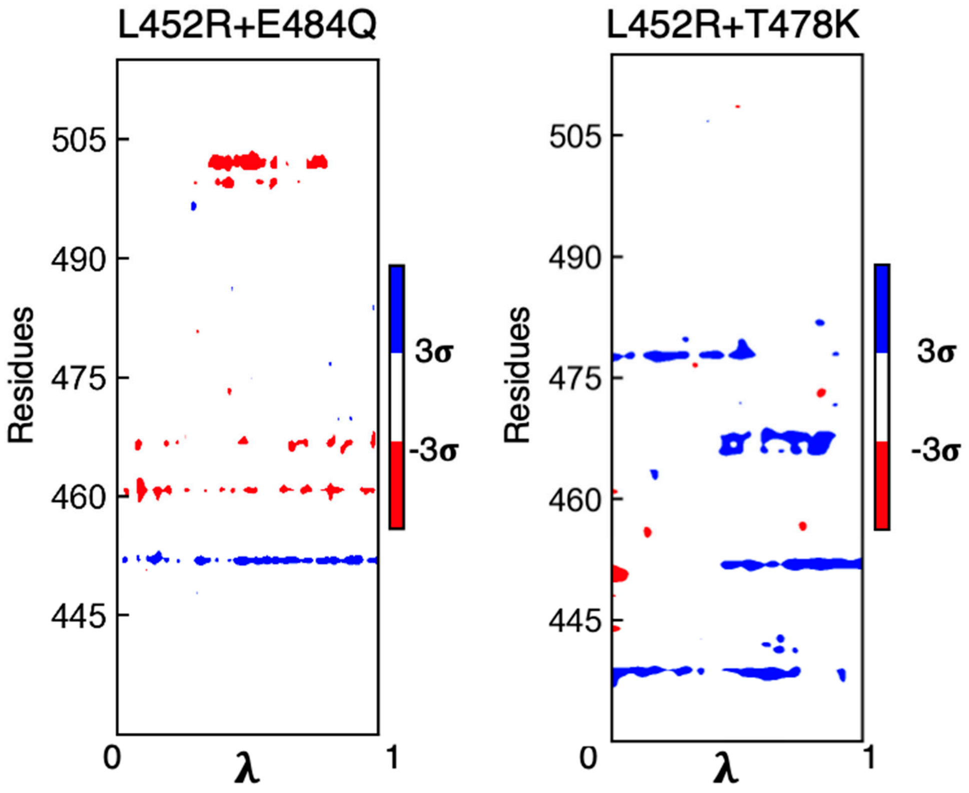

Figure S13. Change in hydration level of residues of mutated RBD in FEP calculations. Hydration levels obtained from MD trajectories were used as references. Only a change with magnitude greater or smaller than three standard deviations would be shown in blue or red respectively.

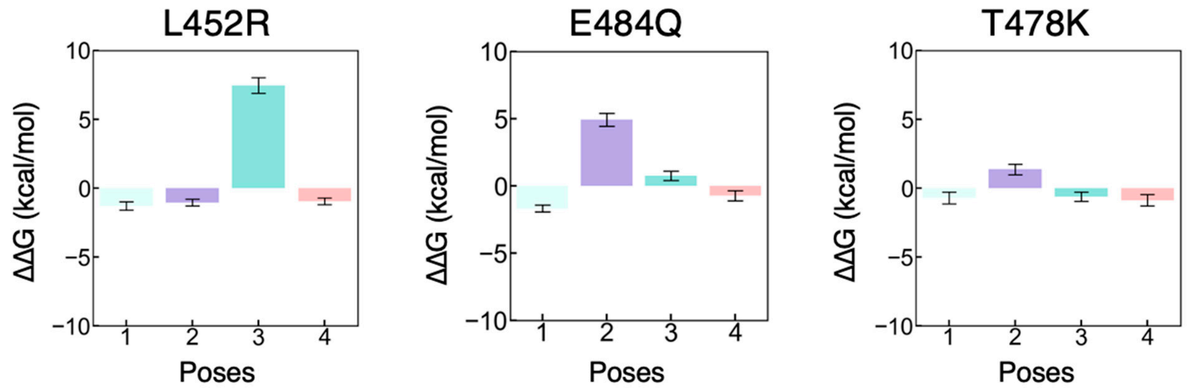

Figure S14. Change in BFE for mAbs concerning RBDs with single mutations. Error bars were the standard errors.

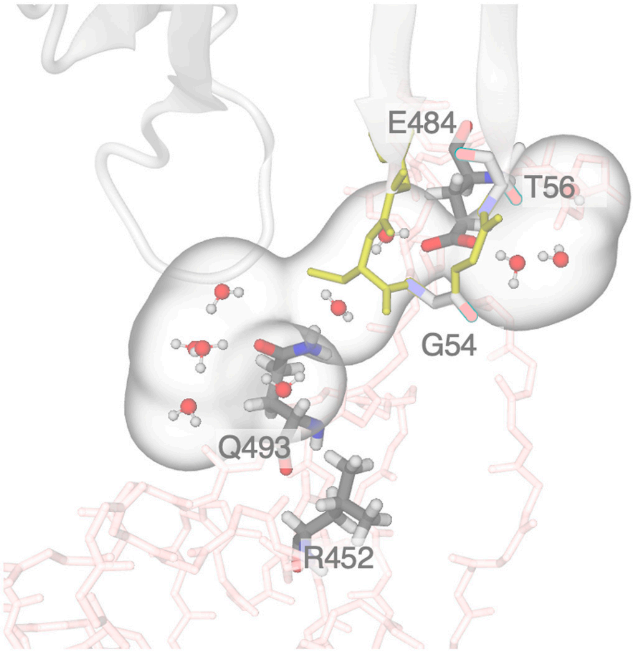

Figure S15. Interaction between COVA2-39 (Pose 2 mAb, white) and RBD_{mut2} (L452R+T478K, pink). Key residues were shown as sticks. Water molecules around Glu484 and Gln493 were shown explicitly as transparent surface. The CDR loop of COVA2-39 containing Gly54 and Thr56 was shown as yellow sticks.

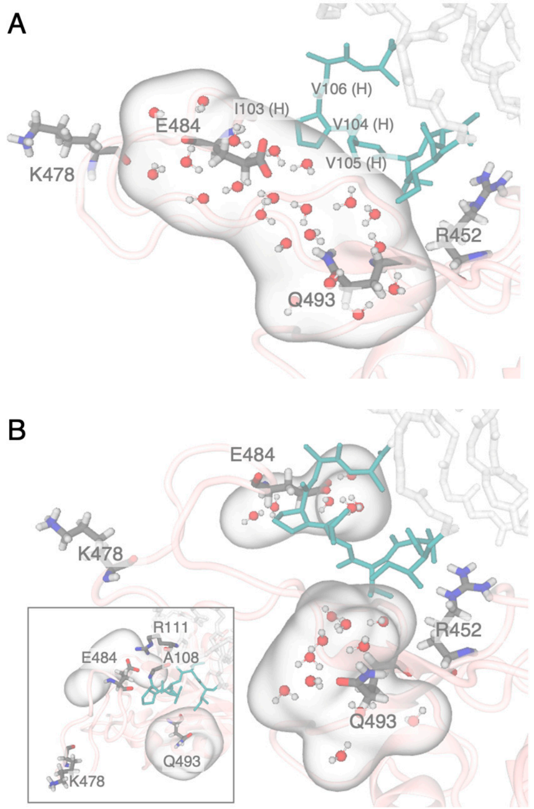

Figure S16. Interaction between P2B-2F6 (Pose 3 mAb, white) and RBD_{mut2} (L452R+T478K, pink). Key residues were shown as sticks. Water molecules around Glu484 and Gln493 were shown explicitly and as transparent surface. The CDR loop of P2B-2F6 containing residues 103-106 was shown as cyan sticks. (A) Snapshot showing that Glu484 and Gln493 of RBD could form water-mediated polar interactions given combinative mutations (L452R+T478K). (B) Snapshot showing the CDR loop could insert in-between Glu484 and Gln493 of RBD. The insert shows interactions between Glu484 of RBD and Arg111 and Ala108 of P2B-2F6.

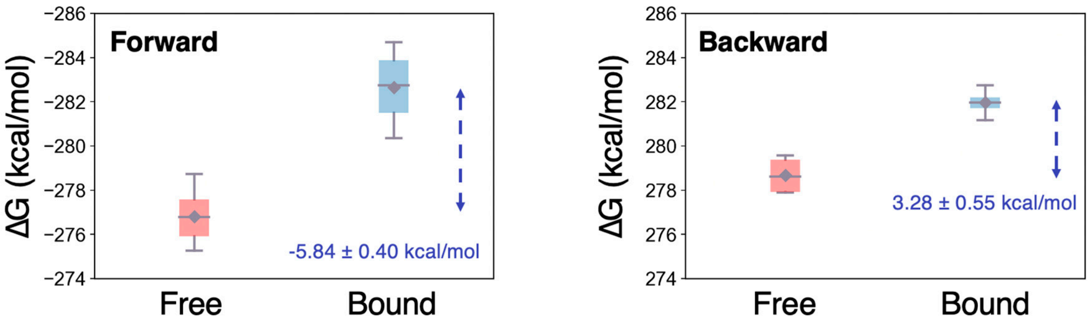

Figure S17. Free energy changes due to combinative mutations in RBD_{mut2} (L452R+T478K) along a forward (left) and a backward (right) alchemical path of 60 windows.

H-bonds	Donor	Acceptor	%
RBD—ACE2 WT	RBD: Gln493 (Side)	ACE2: Glu35 (Side)	46.62
	ACE2: Lys31 (Side)	RBD: Gln493 (Side)	15.78
RBD—ACE2 L452R+E484Q	RBD: Gln493 (Side)	ACE2: Glu35 (Side)	56.55
	ACE2: Lys31 (Side)	RBD: Gln493 (Side)	18.95
RBD—ACE2 L452R+T478K	RBD: Gln493 (Side)	ACE2: Glu35 (Side)	52.08
	ACE2: Lys31 (Side)	RBD: Gln493 (Side)	9.55
**Salt bridges**	**ACE2**	**RBD**	**%**
RBD—ACE2 WT	Lys31	Glu484	8.34
RBD—ACE2 L452R+T478K	Lys31	Glu484	7.83

Table S1. Electrostatic interactions between WT or mutated RBD and ACE2.

	$\Delta\Delta G_{elec}$	$\Delta\Delta G_{vdw}$
L452R	-1.92 ± 0.40	0.36 ± 0.24
T478K	-0.42 ± 0.28	0.29 ± 0.22
L452R+T478K	-1.94 ± 0.49	-4.07 ± 0.30

Table S2. Free energy decomposition analysis. All free energies in kcal/mol.

H-bonds	Donor	Acceptor	%
RBD—COVA2-39 (Pose 2 mAb) WT	mAb: Gly54 (Main)	RBD: Glu484 (Side)	81.53
	mAb: Thr56 (Side)	RBD: Glu484 (Side)	80.20
	mAb: Thr56 (Main)	RBD: Glu484 (Side)	65.99
	RBD: Gln493 (Side)	mAb: Ser31 (Main)	10.04
RBD—P2B-2F6 (Pose 3 mAb) WT	mAb: Lys55 (Side)	RBD: Glu484 (Side)	24.28
	mAb: Lys55 (Side)	RBD: Glu484 (Main)	13.99
RBD—P2B-2F6 (Pose 3 mAb) L452R+E484Q	mAb: Asn33 (Side)	RBD: Gln484 (Main)	12.73
	mAb: Tyr34 (Side)	RBD: Gln484 (Side)	10.87
RBD—P2B-2F6 (Pose 3 mAb) L452R+T478K	mAb: Arg111 (Side)	RBD: Glu484 (Side)	52.67
	mAb: Ala108 (Main)	RBD: Glu484 (Side)	48.07
	mAb: Tyr34 (Side)	RBD: Glu484 (Main)	11.27

Table S3. Electrostatic interactions between WT or mutated RBD and mAbs.

References

1. Eswar, N.; Eramian, D.; Webb, B.; Shen, M.-Y.; Sali, A. Protein Structure Modeling with MODELLER. In *Structural Proteomics: High-Throughput Methods*; Kobe, B., Guss, M., Huber, T., Eds.; Humana Press: Totowa, NJ, USA, 2008; pp. 145–159.
2. Abraham, M.J.; Murtola, T.; Schulz, R.; Páll, S.; Smith, J.C.; Hess, B.; Lindahl, E. GROMACS: High Performance Molecular Simulations through Multi-Level Parallelism from Laptops to Supercomputers. *SoftwareX* **2015**, *1–2*, 19–25. https://doi.org/10.1016/j.softx.2015.06.001.
3. Jorgensen, W.L.; Chandrasekhar, J.; Madura, J.D.; Impey, R.W.; Klein, M.L. Comparison of Simple Potential Functions for Simulating Liquid Water. *J. Chem. Phys.* **1983**, *79*, 926–935. https://doi.org/10.1063/1.445869.
4. Best, R.B.; Zhu, X.; Shim, J.; Lopes, P.E.M.; Mittal, J.; Feig, M.; Mackerell Jr., A.D. Optimization of the Additive CHARMM All-Atom Protein Force Field Targeting Improved Sampling of the Backbone φ, ψ and Side-Chain χ 1and χ 2Dihedral Angles. *J. Chem. Theory Comput.* **2012**, *8*, 3257–3273. https://doi.org/10.1021/ct300400x.
5. Vanommeslaeghe, K.; Hatcher, E.; Acharya, C.; Kundu, S.; Zhong, S.; Shim, J.; Darian, E.; Guvench, O.; Lopes, P.; Vorobyov, I.; et al. CHARMM General Force Field: A Force Field for Drug-like Molecules Compatible with the CHARMM All-Atom Additive Biological Force Fields. *J. Comput. Chem.* **2010**, *31*, 671–690. https://doi.org/10.1002/jcc.21367.
6. Miyamoto, S.; Kollman, P.A. Settle—An Analytical Version of the Shake and Rattle Algorithm for Rigid Water Models. *J. Comput. Chem.* **1992**, *13*, 952–962.
7. Darden, T.; York, D.; Pedersen, L. Particle mesh Ewald: An N log (N) method for Ewald sums in large systems. *J. Chem. Phys.* **1993**, *98*, 10089.
8. Bussi, G.; Zykova-Timan, T.; Parrinello, M. Isothermal-isobaric molecular dynamics using stochastic velocity rescaling. *J. Chem. Phys.* **2009**, *130*, 074101-10.
9. Parrinello, M.; Rahman, A. Polymorphic transitions in single crystals: A new molecular dynamics method. *J.Appl. Phys.* **1981**, *52*, 7182–7190.
10. Humphrey, W.; Humphrey, W.; Dalke, A.; Dalke, A.; Schulten, K.; Schulten, K. VMD: Visual Molecular Dynamics. *J. Mol. Graph. Model.* **1996**, *14*, 33–38.
11. Phillips, J.C.; Braun, R.; Wang, W.; Gumbart, J.; Tajkhorshid, E.; Villa, E.; Chipot, C.; Skeel, R.D.; Kalé, L.; Schulten, K. Scalable Molecular Dynamics with NAMD. *J. Comput. Chem.* **2005**, *26*, 1781–1802. https://doi.org/10.1002/jcc.20289.
12. Hansmann, U.; Okamoto, Y.; Eisenmenger, F. Molecular dynamics, Langevin and hydrid Monte Carlo simulations in a multicanonical ensemble. *Chem. Phys. Lett.* **1996**, *259*, 321–330.
13. Feller, S.E.; Zhang, Y.; Pastor, R.W.; Brooks, B.R. Constant pressure molecular dynamics simulation: The Langevin piston method. *J. Chem. Phys.* **1995**, *103*, 4613–4621.
